# Lower adenoma detection rate in anesthesia assisted colonoscopy: a retrospective study

**DOI:** 10.3389/fonc.2025.1571387

**Published:** 2025-04-10

**Authors:** Chang Cai, Zhihua Xu, Bin Ye

**Affiliations:** Department of Gastroenterology, The Fifth Affiliated Hospital of Wenzhou Medical University and Lishui Municipal Central Hospital, Lishui, China

**Keywords:** colonoscopy, endoscopy, anesthesia assistance, ADR, PDR

## Abstract

**Objectives:**

As anesthesia assisted (AA) colonoscopy becomes increasingly popular, there has been concern about its impact on the quality of colonoscopy examinations. We aimed to clarify the impact of anesthesia assistance on the adenoma detection rate (ADR) and non-adenomatous polyp detection rate (PDR) of colonoscopy.

**Methods:**

We collected data from patients undergoing colonoscopy throughout the year 2023 at our institution, with a total of 16,465 cases identified for potential analysis. After using propensity score matching (PSM) to minimize the influence of other variables on the study outcomes, there were 6,094 cases remaining for analysis in both the AA group and non-AA group, respectively. Then, we compared the ADR and PDR between the two groups and analyzed the colon location and size of adenomas or polyps found in different groups.

**Results:**

The ADR in the non-AA group (36.94%) was significantly higher than that in the AA group (26.40%) (p<0.0001), while there was no statistically significant difference in the PDR between the two groups. AA could also affect the probability of discovering adenomas or polyps in some colon segments, but had no significant effect on the size of the discovered adenomas or polyps. In addition, there were more significant advantages of ADR in the non-AA group among the more experienced endoscopists’ cohort.

**Conclusions:**

Non-AA colonoscopy had a higher ADR, suggesting that while AA may potentially reduce patient stress responses to some extent, it confers few benefits in terms of adenoma detection.

**Clinical Trial Registration:**

https://www.chictr.org.cn/showproj.html?proj=232248.

## 1 Introduction

Colonoscopy is an effective method for evaluating colon and rectal lesions. The use of colonoscopy for early diagnosis and treatment of colorectal tumors, especially colorectal cancer, has been proved to improve disease prognosis (1). To reduce imagined stress reactions and discomfort, lots of patients prefer to choosing anesthesia assisted (AA) colonoscopy recently (2). However, whether it will affect the quality of colonoscopy examination or produce adverse reactions remains to be determined. Some studies have shown that endoscopists administering moderate sedatives increased polyp detection rate (PDR) (3). Conversely, more researchers have found that anesthesia assistance had no significant impact on the quality of colonoscopy and the occurrence of side reactions (4, 5).

In addition to PDR, adenoma detection rate (ADR) is considered one of the most critical quality indicators in colonoscopy (6) and has shown evidence to be an independent predictor of cancer risk in interval colorectal cancer (7). In this study, we used a retrospective approach to evaluate whether AA affects the ADR or PDR of colonoscopy by adjusting for data bias and confounding variables via propensity score matching (PSM).

## 2 Methods

### 2.1 Study population

A total of 22,781 patients who underwent colonoscopy at the Fifth Hospital Affiliated to Wenzhou Medical University from January 1, 2023 to December 31, 2023 were included in the study. Exclusion criteria were as follows: (1) Emergency patients. (2) Patients under 18 years of age. (3) Patients who had been diagnosed with colorectal adenomas. (4) The colonoscopy failed to reach the ileocecal region due to various reasons (e.g. poor bowel preparation). (5) Patients who had undergone colon surgery. (6) Patients with severe colorectal diseases such as inflammatory bowel disease, colorectal malignancy, colorectal metastasis, lymphoma, etc. (7) Patients who underwent colonoscopy for non-routine reasons such as bleeding or rectal foreign bodies. (8) Patients who were unable to tolerate non-AA colonoscopy and switched to AA colonoscopy midway. (9) Incomplete data.

The study was conducted in accordance with the latest version of the Helsinki Declaration, and approved by the Ethics Committee of the Fifth Hospital Affiliated to Wenzhou Medical University in China.

### 2.2 Study procedures

Patients were divided into 2 groups, the non-AA colonoscopy group (non-AA group) and the AA colonoscopy group (AA group), based on whether they underwent AA colonoscopy examinations. The decision to undergo colonoscopy under anesthesia was considered voluntary by the patients. Basic information collected for each group included sex, age, patient type (outpatient or physical examination), endoscopist number, etc. Collected information related to colonoscopy for patients included whether colorectal adenomas were found, whether colorectal polyps were found, the location of adenomas or polyps, the size of adenomas or polyps, bowel prepare score, withdrawal time, etc.

Endoscopists used propofol-lidocaine-fentanyl for sedation anesthesia according to the prescribed AA guidelines, with specific dosages adjusted appropriately based on individual patient conditions, whereas this part of the data was not recorded in the study. Additionally, endoscopists were divided into 3 groups based on their examination experience. The most experienced endoscopists (Group A) were defined as someone performing over 10,000 colonoscopies, and the moderately experienced group (Group B) included those who had performed over 5,000 colonoscopies, while the in-experienced endoscopists (Group C) had performed fewer than 5,000 colonoscopies. In this study, we grouped the baseline data of endoscopists ‘ adenoma detection rates into three categories based on the following thresholds: Group D (adenoma detection rate >35%), including endoscopists A1, A2, A7, B1, and B5, Group E (25% ≤ adenoma detection rate <35%), including endoscopists A5, A6, A9, A10, B2, B3, B4, C1, C2, C3, and C4, while Group F (adenoma detection rate <25%), including endoscopists A3, A4, A8, B6 and C5.

Before colonoscopy screening, all patients underwent a 12-hour fasting and polyethylene glycol (PEG) bowel preparation. Bowel cleanliness was assessed using the Boston Bowel Preparation Scale (BBPS) (8). In brief, BBPS is the sum of scores for the three segments of the colon (right, including the cecum and ascending colon; transverse, including the hepatic and splenic flexures; left, including descending colon, sigmoid colon and rectum), with each segment scored from 0 to 3. The scoring criteria are as follows: 0 = unprepared colon segment with mucosa not seen because of solid stool that cannot be cleared. 1 = portion of mucosa of the colon segment seen, but other areas of the colon segment are not well seen because of staining, residual stool, and/or opaque liquid. 2 = minor amount of residual staining, small fragments of stool, and/or opaque liquid, but mucosa of colon segment is seen well. 3 = entire mucosa of colon segment seen well, with no residual staining, small fragments of stool, or opaque liquid (8). Poor bowel preparation was defined as a total BBPS score <6.

ADR or PDR are defined as the percentage of cases where at least one histologically confirmed adenoma or polyp were detected during endoscopic examination. Please note that polyps in this article only refer to non-adenomatous polyps. Withdrawal time means the actual time from reaching the cecum to withdrawing from the anus. Adenoma/polyp sites include the cecum (ileocecal valve), ascending colon, hepatic flexure, transverse colon, splenic flexure, descending colon, sigmoid colon, and rectum. Adenoma/polyp size is reflected by its maximum diameter. For patients with multiple adenomas/polyps, the site is noted as multiple-site, and the size is represented by the average maximum diameter.

The primary endpoint of the study was to observe whether there was a difference in ADR or PDR between the AA group and the non-AA group, while the secondary endpoint was to analyze whether AA affected the location and/or size of adenomas/polyps discovered.

### 2.3 Statistical analysis

The sample size was based on all data of patients who underwent colonoscopy examination throughout the year 2023 at the Endoscopy Center of the Fifth Hospital Affiliated to Wenzhou Medical University, Lishui, China. The research results were expressed as the number (percentage) of categorical variables and the mean ± standard deviation (SD) of continuous variables. Independent sample t-test and chi square test were used for intergroup continuous variables and categorical variables, respectively. PSM was used to reduce potential confounding effects and balance the differences in baseline characteristics between the two groups. The variables used for matching included age, sex, patient type, qualification level of endoscopists (Group A or B or C), withdrawal time, and BBPS score. We used the nearest neighbor method to match patients in a 1:1 ratio with a matching tolerance of 0.0005. In the propensity matching queue, paired chi square test and paired rank sum test were used to compare paired groups.

The data analysis was analyzed using SPSS software 27.0 (SPSS Inc., Armonk, NY, USA), and two-tailed p values <0.05 were considered statistically significant.

## 3 Results

Among the total 22,781 patients who underwent colonoscopy at the hospital from January 1 to December 31 in the year 2023, data from 16,465 cases were ultimately included for analysis. Based on whether participating in AA, they were divided into the non-AA group (7,322, 44.47%) and the AA group (9,143, 55.53%). The basic characteristics of patients in each group are shown in **Table 1**. It is evident that except for BBPS and withdrawal time, which showed no statistical significance between the two groups, all other variables, including ADR and PDR, had p values less than 0.05. The cecal intubation rate for non-AA colonoscopy was 99.74%, while that for AA colonoscopy was 99.65%. There was no statistically significant difference in cecal intubation rates between the two groups(95% CI 1.00~1.00, p=0.31).

**Table 1:** Patient characteristics before PSM (n=16,465).

	Non-AA^a^ (n=7,322)	AA (n=9,143)	P value
Age (yrs)	54.52 ± 11.77	50.26 ± 11.12	**<0.0001**
18~39	780 (10.65%)	1814 (19.84%)	
40~49	1481 (20.23%)	2455 (26.85%)	
50~59	2506 (34.23%)	2821 (30.85%)	
60~69	1854 (25.32%)	1528 (16.71%)	
70~79	651 (8.89%)	493 (5.39%)	
>79	50 (0.68%)	32 (0.35%)	
Gender			**<0.0001**
Male	3937 (53.77%)	4335 (47.41%)	
Female	3385 (46.23%)	4808 (52.59%)	
Patient type			**<0.0001**
Outpatient	6932 (94.67%)	7768 (84.96%)	
Physical examination	390 (5.33%)	1375 (15.04%)	
Endoscopist group			**<0.0001**
A	4090 (55.85%)	4073 (44.55%)	
B	1630 (22.26%)	1286 (14.07%)	
C	1602 (21.88%)	3784 (41.39%)	
BBPS^b^	7.99 ± 0.71	8.01 ± 0.71	0.0964
Withdrawal time (min)	8.99 ± 1.08	8.99 ± 1.06	0.4179
ADR^c^	2792 (38.13%)	2305 (25.21%)	**<0.0001**
PDR^d^	1406 (19.20%)	1627 (17.80%)	**0.0211**
Endoscopist group			**<0.0001**
D	2134 (29.14%)	2336 (25.55%)	
E	3717 (50.76%)	4247 (46.45%)	
F	1471 (20.09%)	2560 (28.00%)	

a AA, anesthesia assistance/anesthesia assisted.

b BBPS, Boston Bowel Preparation Scale.

c ADR, adenoma detection rate.

d PDR, polyp detection rate. Bold values indicates a statistically significant difference.

**Figure 1:**
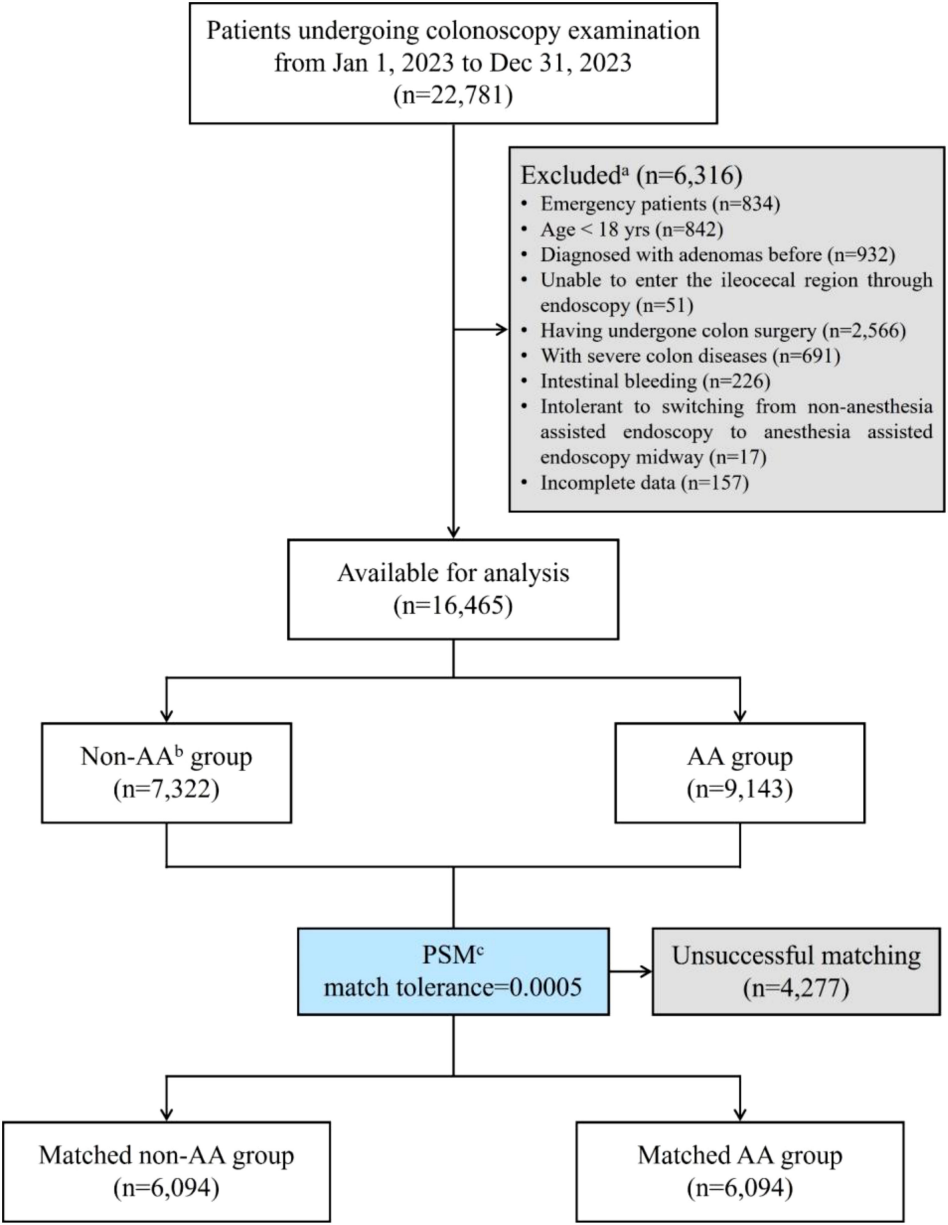
Flow diagram of the study population. ^a^This study conducted stepwise exclusion according to the order of exclusion criteria, so some cases that simultaneously met multiple exclusion criteria were not reflected. ^b^AA, anesthesia assistance or anesthesia assisted. ^c^PSM, propensity score matching.

To investigate whether AA influenced ADR or PDR, PSM with a matching tolerance of 0.0005 was used to eliminate differences between other variables. After matching, there were 6,094 patients remaining for analysis in each group (**Table 2**). The basic steps of data processing have been shown in **Figure 1**. Obviously, apart from the primary observed endpoints of this study, there were no statistically significant differences in other variables between the two groups, indicating good matching. However, the ADR in the non-AA group was still significantly higher than that in the AA group (p<0.0001), while there was no statistically significant difference in PDR between the two groups (p=0.7656).

**Table 2:** Patient characteristics after PSM (n=12,188).

	Non-AA^a^ (n=6,094)	AA (n=6,094)	P value
Age (yrs)	52.90 ± 11.58	52.79 ± 11.71	0.5438
18~39	767 (12.59%)	845 (13.87%)	
40~49	1400 (22.97%)	1450 (23.79%)	
50~59	2158 (35.41%)	2037 (33.43%)	
60~69	1353 (22.20%)	1302 (21.37%)	
70~79	397 (6.51%)	433 (7.11%)	
>79	19 (0.31%)	27 (0.44%)	
Gender			0.1742
Male	3118 (51.17%)	3042 (49.92%)	
Female	2976 (48.83%)	3052 (50.08%)	
Patient type			0.1482
Outpatient	5707 (93.65%)	5746 (94.29%)	
Physical examination	387 (6.35%)	348 (5.71%)	
Endoscopist group			0.1418
A	3498 (57.40%)	3406 (55.89%)	
B	1025 (16.82%)	1099 (18.03%)	
C	1571 (25.78%)	1589 (26.07%)	
BBPS^b^	8.00 ± 0.71	7.99 ± 0.71	0.8159
Withdrawal time (min)	8.99 ± 1.08	8.98 ± 1.06	0.8857
ADR^c^	2251 (36.94%)	1609 (26.40%)	**<0.0001**
PDR^d^	1186 (19.46%)	1172 (19.23%)	0.7656
Endoscopist group			**<0.0001**
D	1697	1927	
E	3087	2602	
F	1310	1565	

a AA, anesthesia assistance/anesthesia assisted.

b BBPS, Boston Bowel Preparation Scale.

c ADR, adenoma detection rate.

d PDR, polyp detection rate. Bold values indicates a statistically significant difference.

**Figure 2:**
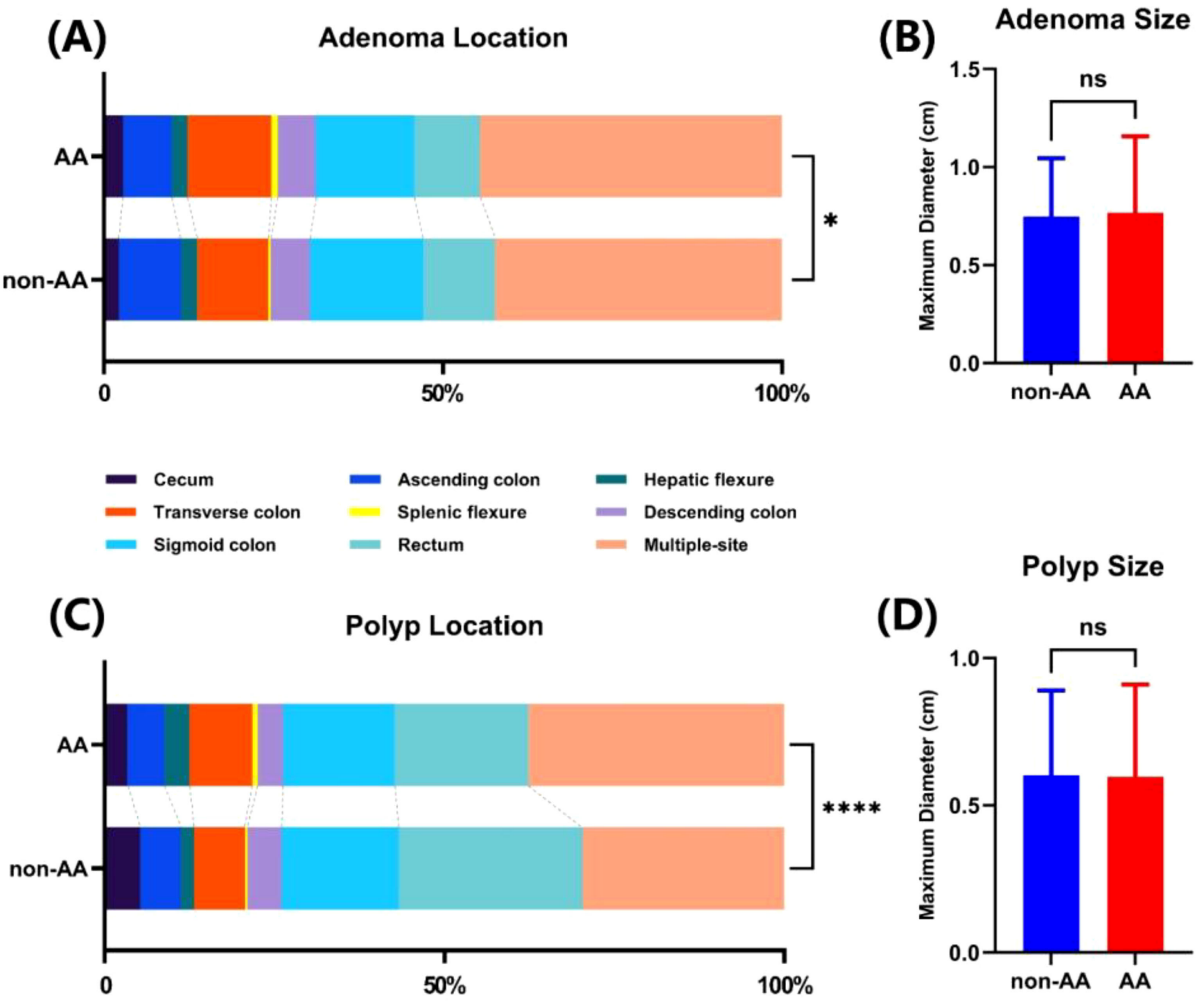
The influence of AA on the discovery location and size of adenomas and polyps. Colonic localization **(A)** and size **(B)** of adenomas found in two groups. Colonic localization **(C)** and size **(D)** of polyps found in two groups. ns, not significant. *p<0.05. ****p<0.0001.

We further analyzed the impact of AA on the location and size of detected adenomas or polyps, as shown in **Figure 2**. There was no statistical difference in the size of adenomas or polyps discovered under anesthesia or not (**Figures 2B, D**). Conversely, colonoscopies performed under anesthesia could detect more transverse colon or multiple adenomas or polyps, while endoscopies without anesthesia were more likely to find adenomas or polyps in the sigmoid colon and rectum (**Figure 2A, C**).

**Figure 3:**
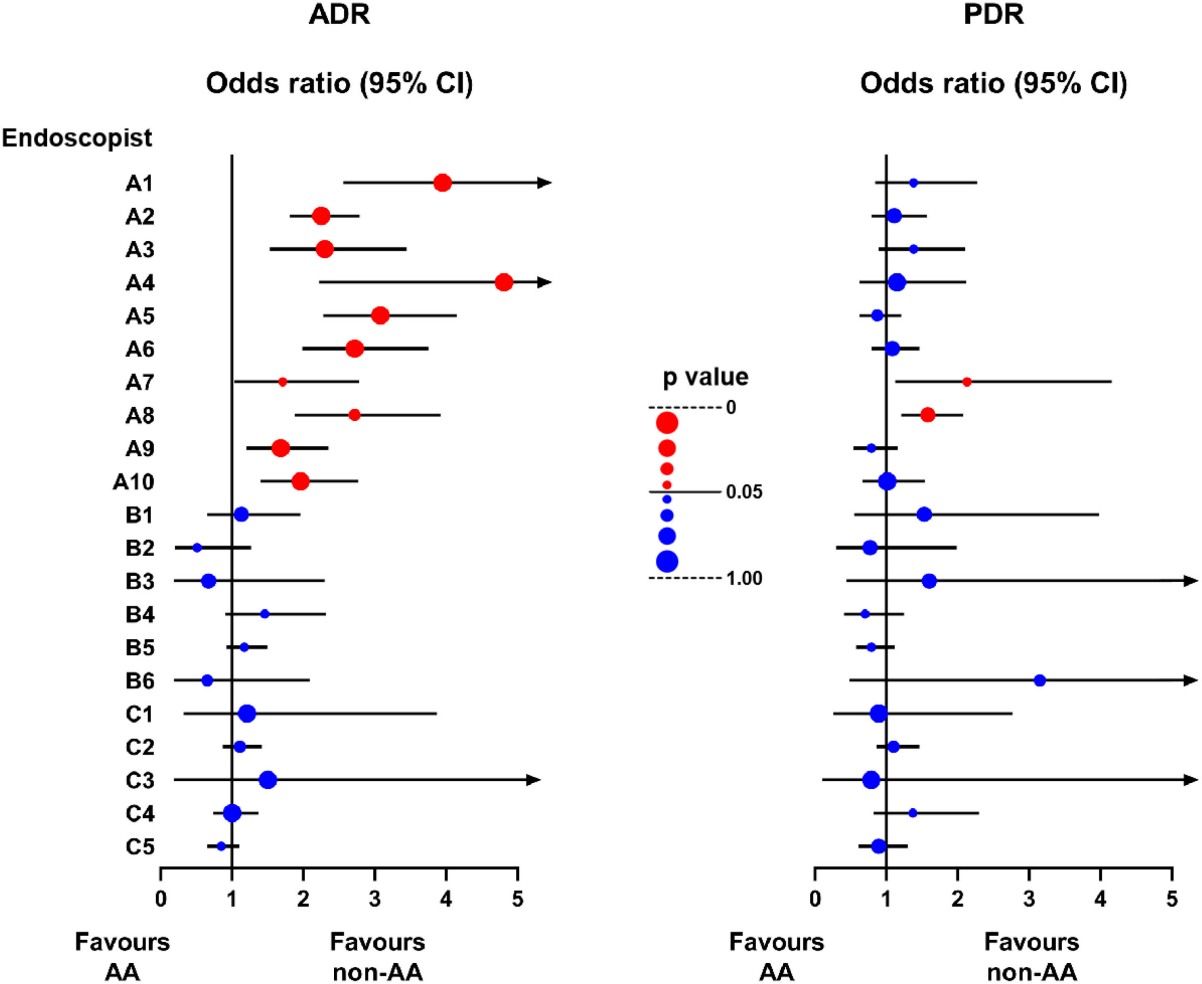
Forest plot of the odds ratio of ADR and PDR between non-AA and AA groups in the queue of each endoscopist after PSM. The line segment consists of odds ratio (OR) (dot) and 95% confidence interval (CI) (line). The color and size of the dots represent whether the p value is statistically significant. Red: p<0.05. Blue: p≥0.05. Larger: closer to 0 or 1.

Additionally, we analyzed the comparison of ADR or PDR between AA and non-AA colonoscopy procedures for each endoscopist after balancing other factors with PSM. As shown in **Figure 3**, the most experienced endoscopists from Group A showed significantly higher ADR in the non-AA group compared to the AA group. Endoscopist No. A4 showed the most remarkable difference between two groups, with the odds ratio (OR) reaching 4.81 (95% CI 2.22~10.30, p<0.0001) (**Supplementary Table 1**). However, no statistically significant difference in ADR between the two conditions had been found in the queue of other less experienced endoscopists. Furthermore, except for a few experienced endoscopists showing higher PDRs in the non-AA group, there were no other statistically significant differences in PDR.

## 4 Discussion

Since the introduction of anesthesia into colonoscopy, there have been numerous studies on whether anesthesia has a meaningful impact on the quality of colonoscopy. Most of them revealed that there was no statistically significant difference in ADR or PDR between AA and non-AA colonoscopies (4, 9, 10), but a study on 63,417 cases indicated a higher ADR for AA colonoscopy (11). Sui et al. (12) found that although no dramatic difference was detected between the two groups (46.3% vs. 45.4%, p>0.05), the adenomas per positive patient (APP) in the non-AA group was higher than that in the AA group (2.00 ± 1.30 vs. 1.76 ± 0.81, p<0.05), suggesting that non-AA colonoscopy could discover a higher number of adenomas to some extent. Besides, a recent cohort study showed that the use of propofol sedation was not associated with improvement in quality indicators related to colonoscopy, but it would increase extra costs by approximately $12,730,496 CAD per 100,000 cases (13). For Zhejiang Province specifically, AA colonoscopy incurs approximately 800 RMB higher costs compared to non-AA colonoscopy. Additionally, anesthetized patients require extra pre-anesthetic evaluations and post-procedural recovery care, which further increases labor costs and places greater strain on healthcare resources. Additionally, patients will spend more time throughout the entire colonoscopy process. Furthermore, AA colonoscopies often require family members to accompany the patient, which may impose an additional burden on the family. Moreover, it has been evidenced that anesthesia was associated with a higher risk of complications such as aspiration, splenic injury, perforation, etc (14). All those researches manifested that AA colonoscopy did not seem to show obvious advantages over direct colonoscopy examination.

In our study, despite initial differences in patients’ baseline information (e.g., age, gender, patient type) between the AA and non-AA groups, no statistical differences were observed after PSM, indicating comparability of the propensity matching queue. The cecal intubation rates were comparable between the non-AA colonoscopy and AA colonoscopy (99.74% vs. 99.65%, p= 0.31). This study found that non-AA colonoscopy had a higher ADR than AA colonoscopy (36.94% vs. 26.40%, p<0.0001), while PDR showed no statistically significant difference (19.46% vs. 19.23%, p=0.7656). It is worth noting again that the PDR referred to in the article refers to non-adenomatous PDR. Interestingly, in the studies of other scholars mentioned above, the ADR of non-AA colonoscopy ranged from 9.4% to 21.2%, whereas in our study, the ADR for the non-AA group exceeded 35% regardless of PSM, remarkably higher than that in other studies, which may be one of the reasons for the differences in conclusions between our study and other studies. And the high ADR in this study was likely to be associated with incomplete historical records in the patient data export system of the hospital. Some patients might not be undergoing their colonoscopy screening for the first time, but their medical history of previously diagnosed or detected adenomas or polyps was unclear or not accurately recorded, resulting in these cases not being successfully excluded. However, this error would not prominently affect patients’ motivation to choose undergoing AA or non-AA colonoscopy. It could be considered that there may be a similar proportion of patients in both AA and non-AA groups who did not received colonoscopy for the first time. Therefore, the comparative results between the two groups in the study should still be accepted statistically significant. Besides, we excluded cases of poor bowel preparation (BBPS<6), which might also lead to higher ADR in our study, as most of other studies did not exclude this group of patients.

To explain the dramatically higher ADR in the non-AA group in our study, one possible reason is that the vast majority of endoscopists involved were used to adjusting patients’ positions as needed during the withdrawal phase of the colonoscopy. Specifically, patients would be required to be supine position when the endoscope was in the cecum to the right half of the transverse colon, or right lateral decubitus position when exploring the left half of the transverse colon and descending colon, or left lateral decubitus position when the endoscope reached the sigmoid and rectum. Since gas is less dense than liquid and tends to accumulate at the higher sections of a cavity, this practice of changing positions can expose the lumen more fully (15), making it easier to observe subtle lesions. Patients under anesthesia have difficulty changing positions during the examination, while non-AA patients can easily change positions with the guidance of the endoscopist and assistant, thus improving the ADR. This conclusion has been confirmed by previous researchers (16–19). It should be pointed out that the specific methods of changing positions in some studies may differ from those in our study, but the objective is the same, hence the conclusions can still be referenced.

Furthermore, the preference for using the water exchange method in non-AA colonoscopy might be another reason for the higher ADR. Fuccio et al. (20) had mentioned that colonoscopies performed with the water exchange method could reduce patient discomfort, enhance bowel cleanliness, and improve ADR. Although this method may prolong the insertion time, it does not increase the withdrawal time (20).

The ADR is also closely related to the withdrawal time of the colonoscope. A withdrawal time of over 6 minutes can detect more adenomas, but whether a withdrawal time of 9 minutes is necessary depends on the operator’s experience (21). In this study, because the withdrawal time of both groups was more than 6 minutes with no statistically significant difference (8.99 ± 1.08 vs. 8.98 ± 1.06, p=0.8857), the possibility of the withdrawal time affecting ADR can be ruled out.

In addition, we also found that AA or non-AA affected the intestinal location of detected adenomas or polyps, but did not impact the size. Non-AA colonoscopy had more advantages in finding lesions of the sigmoid colon and rectum. A possible reason is that compared to patients undergoing anesthesia, conscious patients can effectively control the anal sphincter, prevent injected gas or water from overflowing from the intestine, and maintain a clear field of view of the sigmoid and rectal segments, which is beneficial for ADR and PDR. On the contrary, AA colonoscopy was beneficial for detecting adenomas or polyps in the transverse colon and had certain advantages in discovering multiple lesions, which may be due to the fact that anesthetized patients do not provide feedback on discomfort or pain during the examination process, resulting in endoscopists performing examinations on these patients with greater force to determine adenomas or polyps in remote areas. This also suggests that subsequent prospective trials should strictly implement quality control to exclude differences in results caused by differences in the operation of endoscopists.

Our study further compared the ADR of each endoscopist in both AA and non-AA groups. Almost all endoscopic experts showed apparently higher ADRs in the non-AA group, which may be related to their proficiency in conducting non-AA colonoscopy and accurately guiding patients through position changes. For those less experienced endoscopists, the presence or absence of anesthesia did not affect their ability to detect adenomas or polyps. However, we also found that some endoscopists with relatively higher adenoma detection rates despite lower case volumes performed equally effectively in both sedated and non-sedated colonoscopies. Notably, as mentioned earlier, some patients who were not undergoing colonoscopy for the first time might not have been effectively excluded, and those patients were likely to request more experienced endoscopists for their procedures, leading to differences in the prevalence of adenomas or polyps among the patient groups handled by endoscopists of varying experience levels, which potentially contributes to the currently observed results.

There are several limitations in our study. Firstly, as a single center retrospective study, bias is inevitable. Although PSM was used to eliminate the influence of some variables, there are still other unrecorded confounding factors. Specifically, due to incomplete information records, we were unable to reliably collect multiple factors that may affect ADR or PDR, such as family history of colorectal cancer, smoking history, device version of colonoscopy, whether image enhancement was performed during the examination process, and the number of endoscopic images per case. And this is one of the reasons why regression analysis was not conducted in the study. In addition, retrospective studies have also made it difficult for us to confirm whether patients chose to undergo either AA or non-AA colonoscopy scans entirely out of their own will, and the patient’s tolerance to each examination method is also unknown, which requires further prospective trials to be standardized.

Secondly, although the operational level of endoscopists has been roughly stratified, because of the large number of endoscopists, there are also differences in the habits and professional level of colonoscopy examination among the same group of endoscopists. For example, as shown in **Supplementary Table 1**, despite being assigned to Group A because of his extensive experience, the ADR of NO. A8 endoscopist was much lower than other endoscopists in the same group, indicating that the stratification criteria for endoscopists in the study need further improvement. Furthermore, although NO. A4 endoscopist had performed over 10,000 endoscopic procedures in history, his total number of colonoscopy examinations performed in the year 2023 was relatively low, so his high OR may not necessarily have reference value.

In summary, our retrospective study found that non-AA colonoscopy had a higher ADR than AA colonoscopy. In the future, prospective multi-center studies should be conducted to further clarify the impact of anesthesia assistance on ADR and PDR, and additional attention should be paid to patient tolerance during and after the examination, the incidence of adverse events during and after the examination, and whether patients need to bear additional costs. Moreover, strict and scientific regulations should be established for the selection and grouping of patients and the endoscopists involved in the study.

## Data Availability

The raw data supporting the conclusions of this article will be made available by the authors, without undue reservation.

## References

[1] Pan J Xin L Ma YF Hu LH Li ZS. Colonoscopy reduces colorectal cancer incidence and mortality in patients with non-malignant findings: A meta-analysis. Am J Gastroenterol. (2016) 111:355–65. doi: 10.1038/ajg.2015.418 PMC4820666 26753884

[2] Liang M Zhang X Xu C Cao J Zhang Z. Anesthesia assistance in colonoscopy: impact on quality indicators. Front Med. (2022) 9:872231. doi: 10.3389/fmed.2022.872231 PMC9326494 35911411

[3] Chen C-W Chiu C-T Su M-Y Lin C-J Hsu C-M Lim S-N. Factors associated with polyp detection during colonoscopy: A retrospective observational study. Kaohsiung J Med Sci. (2019) 35:572–7. doi: 10.1002/kjm2.12090 PMC11900736 31162814

[4] Bannert C Reinhart K Dunkler D Trauner M Renner F Knoflach P. Sedation in screening colonoscopy: impact on quality indicators and complications. Am J Gastroenterol. (2012) 107:1837–48. doi: 10.1038/ajg.2012.347 23147522

[5] Krigel A Patel A Kaplan J Kong X-F Garcia-Carrasquillo R Lebwohl B. Anesthesia assistance in screening colonoscopy and adenoma detection rate among trainees. Digestive Dis Sci. (2019) 65:961–8. doi: 10.1007/s10620-019-05820-2 31485995

[6] Ruiz-Rebollo ML Alcaide-Suárez N Burgueño-Gómez B Antolin-Melero B Muñoz-Moreno Mª F Alonso-Martín C. Adenoma detection rate and cecal intubation rate: Quality indicators for colonoscopy. Gastroenterol Hepatol. (2019) 42:253–5. doi: 10.1016/j.gastrohep.2018.05.005 29880415

[7] Kaminski MF Regula J Kraszewska E Polkowski M Wojciechowska U Didkowska J. Quality indicators for colonoscopy and the risk of interval cancer. N Engl J Med. (2010) 362:1795–803. doi: 10.1056/NEJMoa0907667 20463339

[8] Lai EJ Calderwood AH Doros G Fix OK Jacobson BC. The Boston bowel preparation scale: a valid and reliable instrument for colonoscopy-oriented research. Gastrointestinal Endoscopy. (2009) 69:620–5. doi: 10.1016/j.gie.2008.05.057 PMC2763922 19136102

[9] Zhao S Deng X-l Wang L Ye J-w Liu Z-y Huang B. The impact of sedation on quality metrics of colonoscopy: a single-center experience of 48,838 procedures. Int J Colorectal Dis. (2020) 35:1155–61. doi: 10.1007/s00384-020-03586-y 32300884

[10] Khan F Hur C Lebwohl B Krigel A. Unsedated colonoscopy: impact on quality indicators. Digestive Dis Sci. (2020) 65:3116–22. doi: 10.1007/s10620-020-06491-0 32696236

[11] Zhang Q Dong Z Jiang Y Zhan T Wang J Xu S. The impact of sedation on adenoma detection rate and cecal intubation rate in colonoscopy. Gastroenterol Res Practice 2020. (2020) p:1–8. doi: 10.1155/2020/3089094 PMC7758148 33381166

[12] Sui Y Wang Q Chen HH Lu JH Wen Q Wang ZZ. Comparison of adenoma detection in different colorectal segments between deep-sedated and unsedated colonoscopy. Sci Rep. (2022) 12:15356. doi: 10.1038/s41598-022-19468-y 36097050 PMC9468171

[13] Rahman S Cipriano LE McDonald C Cocco S Hindi Z Chakraborty D. Propofol sedation does not improve measures of colonoscopy quality but increase cost - findings from a large population-based cohort study. EClinicalMedicine. (2024) 70:102503. doi: 10.1016/j.eclinm.2024.102503 38495522 PMC10940905

[14] Cooper GS Kou TD Rex DK. Complications following colonoscopy with anesthesia assistance: a population-based analysis. JAMA Intern Med. (2013) 173:551–6. doi: 10.1001/jamainternmed.2013.2908 PMC3987111 23478904

[15] Wilson A Saunders BP. Position change during colonoscopy: the oldest and best trick in the book. Gastrointest Endosc. (2015) 82:495–6. doi: 10.1016/j.gie.2015.03.1987 26279350

[16] East JE Bassett P Arebi N Thomas-Gibson S Guenther T Saunders BP. Dynamic patient position changes during colonoscope withdrawal increase adenoma detection: a randomized, crossover trial. Gastrointest Endosc. (2011) 73:456–63. doi: 10.1016/j.gie.2010.07.046 20950801

[17] Lee SW Chang JH Ji JS Maeong IH Cheung DY Kim JS. Effect of dynamic position changes on adenoma detection during colonoscope withdrawal: A randomized controlled multicenter trial. Am J Gastroenterol. (2016) 111:63–9. doi: 10.1038/ajg.2015.354 26526085

[18] Zhao SB Wan H Li ZS Bai Y. Is dynamic position changes an effective way to improve the adenoma detection rate? Am J Gastroenterol. (2016) 111:1359–61. doi: 10.1038/ajg.2016.195 27580783

[19] Li P Ma B Gong S Zhang X Li W. Effect of dynamic position changes during colonoscope withdrawal: a meta-analysis of randomized controlled trials. Surg Endosc. (2021) 35:1171–81. doi: 10.1007/s00464-020-07483-x 32128607

[20] Fuccio L Frazzoni L Hassan C La Marca M Paci V Smania V. Water exchange colonoscopy increases adenoma detection rate: a systematic review with network meta-analysis of randomized controlled studies. Gastrointest Endosc. (2018) 88:589–597.e511. doi: 10.1016/j.gie.2018.06.028 29981753

[21] Barclay RL. Colonoscopy withdrawal: it takes time to do it well. Gastrointest Endosc. (2017) 85:1281–3. doi: 10.1016/j.gie.2017.03.007 28522017

