# Supplementary figures and images for: Lower adenoma detection rate in anesthesia assisted colonoscopy: a retrospective study
Source: Front Oncol. 2025 Apr 10;15:1571387. doi: 10.3389/fonc.2025.1571387 (PMC12018242; doi:10.3389/fonc.2025.1571387)

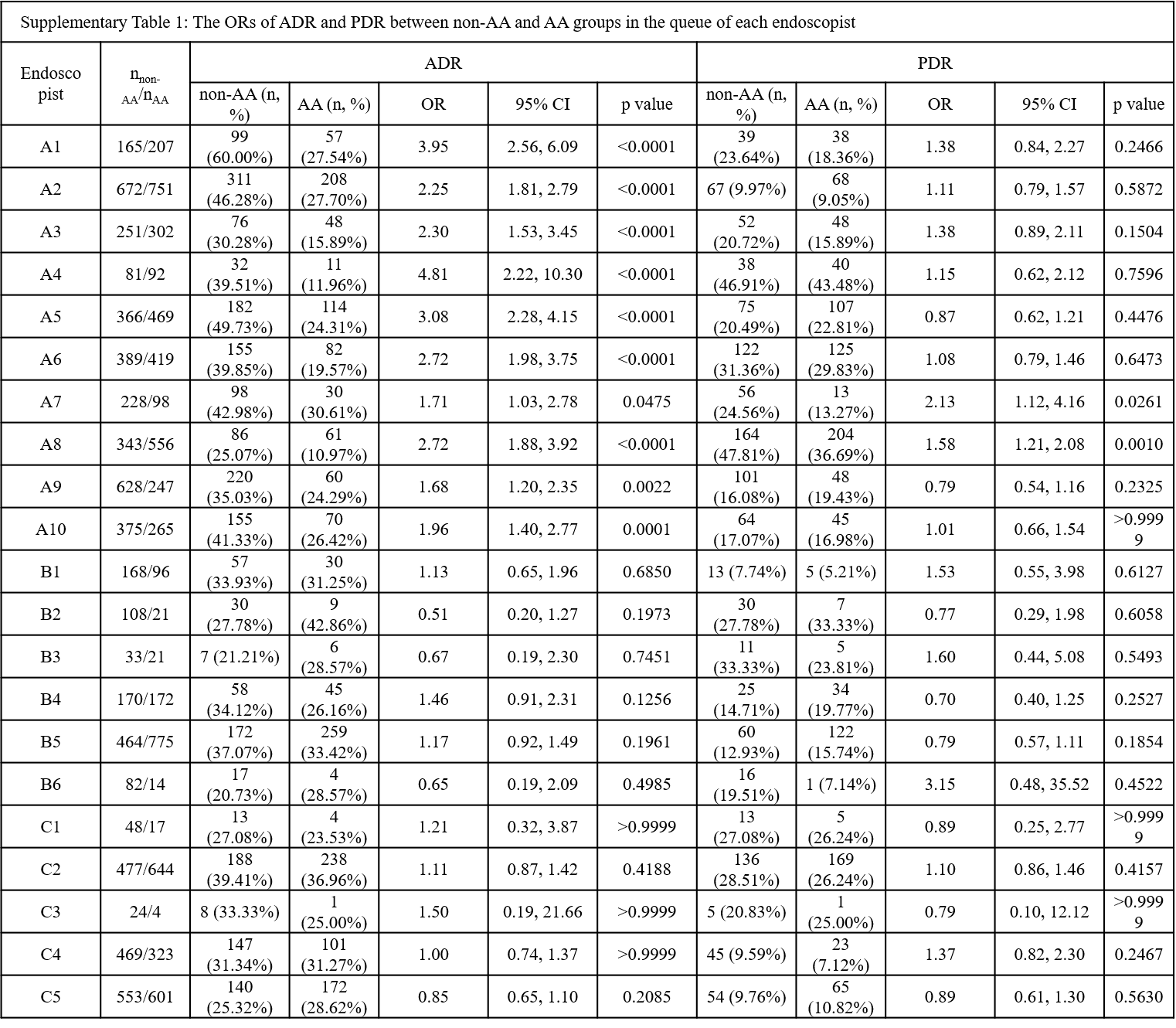

Supplement: Supplementary file 1 [file SupplementaryFile1.png]
